# Decreased Expression of Pulmonary Homeobox NKX2.1 and Surfactant Protein C in Developing Lungs That Over-Express Receptors for Advanced Glycation End-Products (RAGE)

**DOI:** 10.3390/jdb11030033

**Published:** 2023-07-15

**Authors:** Derek M. Clarke, Katrina L. Curtis, Ryan A. Wendt, Brendan M. Stapley, Evan T. Clark, Nathan Beckett, Kennedy M. Campbell, Juan A. Arroyo, Paul R. Reynolds

**Affiliations:** Lung and Placenta Laboratory, Department of Cell Biology and Physiology, Brigham Young University, Provo, UT 84602, USA

**Keywords:** RAGE, surfactant, lung, development

## Abstract

Receptors for advanced glycation end-products (RAGE) are multi-ligand cell surface receptors of the immunoglobin superfamily prominently expressed by lung epithelium. Previous experiments demonstrated that over-expression of RAGE by murine alveolar epithelium throughout embryonic development causes neonatal lethality coincident with significant lung hypoplasia. In the current study, we evaluated the expression of NKX2.1 (also referred to as TTF-1), a homeodomain-containing transcription factor critical for branching morphogenesis, in mice that differentially expressed RAGE. We also contextualized NKX2.1 expression with the abundance of FoxA2, a winged double helix DNA binding protein that influences respiratory epithelial cell differentiation and surfactant protein expression. Conditional RAGE over-expression was induced in mouse lung throughout gestation (embryonic day E0–18.5), as well as during the critical saccular period of development (E15.5–18.5), and analyses were conducted at E18.5. Histology revealed markedly less lung parenchyma beginning in the canalicular stage of lung development and continuing throughout the saccular period. We discovered consistently decreased expression of both NKX2.1 and FoxA2 in lungs from transgenic (TG) mice compared to littermate controls. We also observed diminished surfactant protein C in TG mice, suggesting possible hindered differentiation and/or proliferation of alveolar epithelial cells under the genetic control of these two critical transcription factors. These results demonstrate that RAGE must be specifically regulated during lung formation. Perturbation of epithelial cell differentiation culminating in respiratory distress and perinatal lethality may coincide with elevated RAGE expression in the lung parenchyma.

## 1. Background

Pulmonary development involves highly ordered and complex stages. The formation of the lungs occurs relatively early during embryogenesis, as its origins are observed around embryonic day 9.5 (E9.5) in the mouse. The initial stage of murine pulmonary development involves the establishment of primordial lung buds that undergo branching to form the lobar bronchi, culminating at approximately E11.0. These events are followed by the pseudoglandular stage of development which spans from E11.5 to E16.5. This stage is associated with extensive branching and budding of the lobar bronchi, which forms the intrapulmonary conducting and peripheral lung airways. The pseudoglandular stage is also characterized by the differentiation of primitive epithelium into cell types such as ciliated columnar epithelium, non-ciliated Club cells, and goblet cells that will line the future conducting airways. This period of morphogenesis also coincides with distal parenchymal cell differentiation of alveolar Type I (ATI) and II (ATII) cells that specialize the future respiratory compartment. ATI cells emerge as distal parenchymal cells flatten, thin, and spread, while ATII parenchymal cells remain cuboidal and acquire the capacity to synthesize surfactant [1,2]. The pseudoglandular period is followed by the canalicular (E16.5 to E17.5) and saccular (E17.5 to postnatal (PN) day 4) stages of lung development. These last stages are notably accompanied by dilations of terminal acinar tubules to form alveolar saccules. Differentiation of epithelial cells to form larger alveolar sacs and thinner mesenchyme is then seen in the latter period of the saccular stage and into the alveolar stage, characterized by surface area enhancement and vascular remodeling of capillary networks necessary for gas exchange [3,4,5].

The receptor for advanced glycation end-products (RAGE) is expressed by diverse tissues throughout the body but is most abundantly detected in the lung [6]. As a member of the immunoglobin superfamily of cell surface receptors, expression is observed in numerous cell types, including fibroblasts, macrophages, smooth muscle, endothelium, and epithelium [4,7,8,9,10]. RAGE is centrally involved in a host of signaling networks where ligands engage an extracellular ligand-engaging V region-like domain and perpetuate intracellular signaling via a short but highly charged cytoplasmic domain [4]. Pulmonary RAGE expression tends to dominate on the membranes of differentiated ATI cells, while expression by ATII cells is less abundant [4,6]. We previously demonstrated that RAGE expression tends to have temporal and spatial constraints during lung development and that genetic up-regulation of RAGE leads to pulmonary simplification [3,4,11]. Such simplification when RAGE is genetically induced involves interplay between proliferation and apoptosis during the differentiation of alveolar cell types.

NKX2.1 serves a fundamental role during lung morphogenesis. This 43 kDa homeodomain-containing transcription factor centrally contributes to the growth and differentiation of lung epithelial cells. As an early detectable marker of pulmonary morphogenesis, its expression is restricted to differentiating epithelium, where it cooperates with a host of other transcriptional regulators in order to direct normal lung development [12,13,14]. For instance, NKX2.1 often cooperates with FoxA2 during the induction of target genes that function during morphogenesis and maintenance of pulmonary function [15]. FoxA2 is a member of the forkhead (Fox) family of transcription factors which, along with FoxA1, is expressed with NKX2.1 by respiratory epithelial cells [13].

Because RAGE over-expression during development causes pulmonary hypoplasia with parenchymal simplification, we hypothesized that RAGE over-expression would decrease NKX2.1, FoxA2, and surfactant protein C. Intriguingly, the loss of RAGE expression during development in RAGE-null animals results in an indistinguishable lung phenotype when compared to controls [4,16]. Even though it is probable that parallel signaling pathways compensate for the absence of RAGE, studies that identify the effects of marked up-regulation of RAGE during development are rare. As such, testing our hypothesis required the use of a pulmonary epithelial-cell-specific transgenic mouse wherein RAGE was induced via a Tet-inducible system. Our results demonstrate that RAGE augmentation results in pulmonary dysmorphogenesis that coincides with diminished expression of NKX2.1 and FoxA2 and eventual compromise of the surfactant secreting capacity by alveolar epithelium.

## 2. Materials and Methods

### 2.1. Animals and Tissue Preparation

All mice were in a C57Bl/6 background. Controllable RAGE over-expression was accomplished by mating two transgenic lines to create mice that over-express RAGE via the doxycycline (dox)-inducible mechanism as described previously [3,4]. PCR genotyping determined existence of transgenes from tail biopsies as described beforehand. Eight time-mated pregnant mice were provided dox food (625 mg/kg; Invigo Teklad, St. Louis, MO, USA) from before conception until E18.5 or during various periods were indicated. Lungs from double-transgenic and littermate non-transgenic controls (from at least 8 different pups each) were procured at E18.5 and processed, embedded in paraffin, then sectioned as already described [3,4]. Additionally, E18.5 lungs were removed from pups (from at least 8 different TG and control pups) and total homogenates were subjected to immunoblotting. All mice were housed and used in accordance with a breeding and experimental protocol approved by the Institutional Animal Care and Use Committee at Brigham Young University (21-0202 and 21-0203) approved on 16 March 2021.

### 2.2. Histology and Immunohistochemistry

Lungs from control and double-transgenic mice were processed and embedded in paraffin. Resulting lung sections were first stained using hematoxylin and eosin to visualize basic morphology. Additional sections were also characterized for the expression of cell-specific markers via immunohistochemistry. The following antibodies were employed: Rabbit anti-TTF-1 at 1:1000 (Seven Hills Bioreagents, Cincinnati, OH, USA), Rat anti-RAGE at 1:1000 (MAB1179 R&D systems, Minneapolis, MN, USA), and mouse anti-smooth muscle actin (A5228 Sigma-Aldrich, St. Louis, MO, USA). Fluorescent secondary antibodies were Donkey anti-Mouse IgG Alexa Flour 647 at 1:200 (Invitrogen A-31571, Carlsbad, CA, USA), Goat anti-Rabbit IgG Alexa Flour 488 at 1:200 (A-11034 Invitrogen), and Donkey anti-Rat IgG Alexa Flour 568 at 1:200 (ab175708 Abcam, Boston, MA, USA), and the 4′,6-diamidino-2-phenylindole (DAPI) counterstain was used at 1:2000 (D44462 Sigma-Aldrich). Stained sections were imaged using the Olympus BX51 microscope and Olympus CellSens Standard 3.1).

### 2.3. Protein Analysis

Whole lung lysates from control and double-transgenic mice were homogenized in radioimmunoprecipitation assay (RIPA) supplemented with protease inhibitors (ThermoScientific, Pittsburg, PA, USA). The concentrations of the protein samples were obtained using a BCA Protein Assay Kit (ThermoScientific). Protein samples (10 μg each) were separated on a 10% SDS-PAGE gel and then transferred to a nitrocellulose membrane that was blocked with 5% nonfat milk and separately incubated with the following primary antibodies: Rabbit anti-TTF-1 at 1:1000 (Seven Hills Bioreagents, Cincinnati, OH, USA), Rabbit anti-Pro-SPC at 1:5000 (Seven Hills Bioreagents), rabbit anti-Mat-Sp-C at 1:5000 (Seven Hills Bioreagents), rabbit anti-Mat-Sp-B at 1:5000 (Seven Hills Bioreagents), and Rabbit anti-Rat Foxa2 at 1:1000 (Seven Hills Bioreagents). We used mouse anti-beta Actin (Santa Cruz Biotechnology sc-81178, Santa Cru, CA, USA) as a loading control at 1:1000. Membranes were then incubated overnight at 4 °C with the corresponding secondary antibodies: IRDye^®^ 680RD Donkey anti-Goat IgG at 1:2500 (LiCor 926-68074) and imaged using a Li-Cor Odyssey machine and quantified using image studio. Figures presented are representative of three different immunoblotting experiments performed in quadruplicate.

### 2.4. Statistical Analysis

Mean values ± S.D. from at least six animals per group were assessed by one- and two-way analysis of variance (ANOVA). When ANOVA indicated significant differences, Student *t*-tests were used with Bonferroni correction for multiple comparisons. Results are representative and those with *p* values < 0.05 were considered significant. Statistical analysis was performed with GraphPad Prism 7.0.

## 3. Results

### 3.1. RAGE Over-Expression Caused Pulmonary Simplification

**Figure 1:**
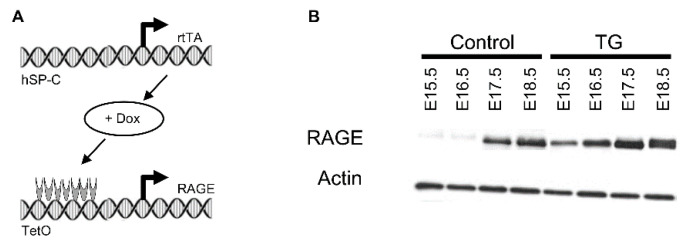
(**A**) Doxycycline (Dox)-inducible expression of RAGE in transgenic (TG) mice required a reverse tetracycline transactivator (rtTA) localized to respiratory epithelial cells via the human surfactant protein (hSP)-C promoter. The rtTA complexed with Dox and activated Tet-inducible RAGE expression. (**B**) Representative immunoblotting revealed increased expression of RAGE in RAGE TG mice fed Dox compared to Dox-exposed control animals. Actin was used as a loading control.

**Figure 2:**
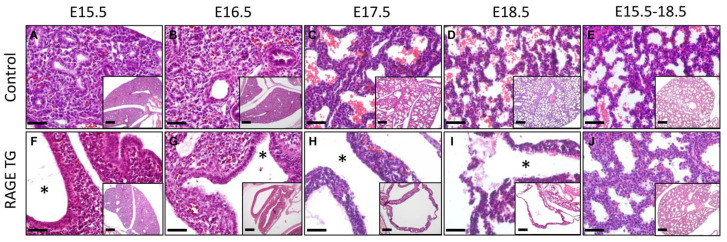
Hematoxylin and eosin staining revealed marked pulmonary simplification was observed in RAGE TG pup lungs compared to controls following RAGE induction from E0–15.5 (**A**,**F**), E0–16.5 (**B**,**G**), E0–17.5 (**C**,**H**), E0–18.5 (**D**,**I**), and E15.5–18.5 (**E**,**J**). Each lung sample from RAGE TG mice with dox administration since conception (**F**–**I**) contained marked tissue reduction (*) with only sporadic areas of distal lung parenchyma. While significant lung hypoplasia resulted from RAGE over-expression from conception, an intermediate phenotype that contained abundant parenchymal tissue prevailed when over-expression occurred from E15-5–E18-5 (**J**). Main image scale bars represent 50 μm and inset image scale bars represent 200 μm.

We have previously published qPCR and immunoblotting confirmation that this transgenic mouse model increased RAGE expression in the embryonic lung [4]. Furthermore, we have previously published details regarding diminished lung weight and lung translucency in TG mice compared to controls [4]. Pregnant dams were exposed to doxycycline (dox) from conception and lungs from pups were isolated at E15.5, E16.5, E17.8, or E18.5. Immunoblotting confirmed RAGE up-regulation in TG mouse lungs compared to controls (Figure 1). Pup lungs were also isolated from dams that were exposed to dox from E15.5 through E18.5 only. Lung sections stained with H&E for histological evaluation revealed extensive morphological alterations in dox-fed double-transgenic mice compared to dox-fed non-transgenic controls (Figure 2). RAGE over-expression through the pseudoglandular and canalicular periods resulted in large dilations devoid of tissue (Figure 2, E15.5 and E16.5). Markedly less lung parenchyma was evident through the saccular period (Figure 2, E17.5 and E18.5), which is a period when primitive respiratory compartments expand and differentiate. We observed persistently thickened respiratory units with increased interstitial cellularity in the E18.5 lung when dox was only administered from E15.5 through E18.5 (Figure 2). Wild-type control and non-transgenic mice were not different when fed dox at any of the time points described.

### 3.2. NKX2.1 Was Down-Regulated in RAGE-Transgenic Mice

**Figure 3:**
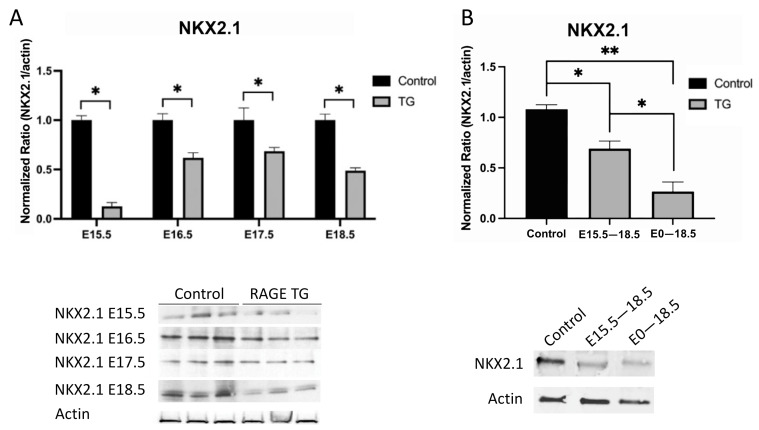
(**A**) Immunoblotting revealed decreased NKX2.1 expression in RAGE TG mice compared to controls when RAGE was overexpressed from E0–15.5, E0–16.5, E0–17.5, or E0–18.5. * *p* ≤ 0.05; blots were densitometrically normalized to β-actin and images are representative of three different immunoblotting experiments performed in quadruplicate. (**B**) Immunoblotting revealed decreased NKX2.1 expression in RAGE TG mice compared to controls when RAGE was overexpressed from E0–18.5 or from E15.5–18.5. * *p* ≤ 0.05 and ** *p* ≤ 0.01; blots were densitometrically normalized to β-actin and images are representative of three different immunoblotting experiments performed in quadruplicate.

**Figure 4:**
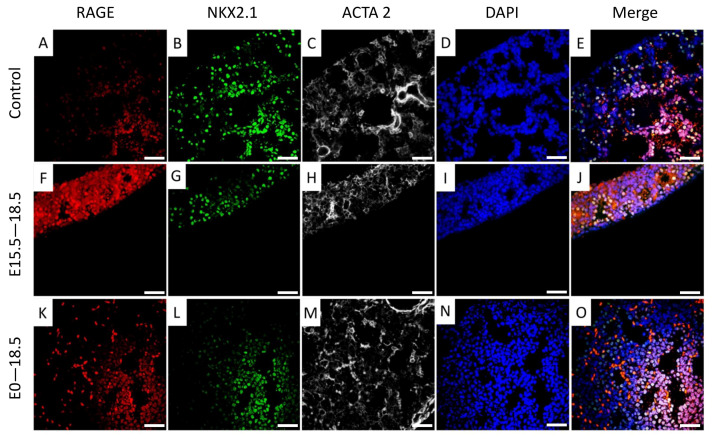
Immunofluorescent staining revealed increased RAGE expression in TG mouse lungs ((**F**,**K**) vs. **A**) and diminished localization of NKX2.1 ((**G**,**L**) vs. **B**) and alpha smooth muscle actin (ACTA 2; (**H**,**M**) vs. **C**). DAPI counterstained images (**D**,**I**,**N**) and merged images that contains RAGE, NKX2.1, ACTA 2, and DAPI (**E**,**J**,**O**) are also depicted. All images are at 400× original magnification and scale bars represent 50 μm.

Protein lysates from pup lungs were isolated daily starting at E15.5 and blotted for NKX2.1. A representative blot for NKX2.1 is shown in Figure 3A. In TG mouse lungs that over-express RAGE (not shown), we discovered significantly less NKX2.1 expression when dox was administered from conception through each of the daily time points from E15.5 to E18.5 (Figure 3A). We further identified differential NKX2.1 expression when dams were exposed to dox from E15.5 through E18.5 (Figure 3B). Although NKX2.1 expression was lowest in pups exposed to dox throughout gestation (E0–E18.5), an intermediate yet still significantly lower level of NKX2.1 expression was detected with dox availability from E15.5–18.5 (Figure 3B). Qualitative immunofluorescent staining revealed diminished NKX2.1 and enhanced RAGE expression in transgenic mice exposed to dox from E0–E18.5 or E15.5–18.5 (Figure 4).

### 3.3. FoxA2 Was Down-Regulated in RAGE-Transgenic Mice

**Figure 5:**
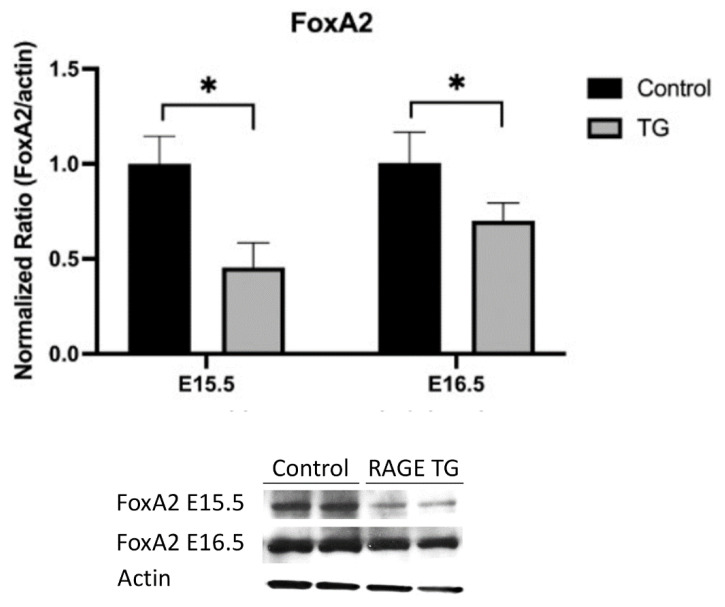
Immunoblotting revealed decreased FoxA2 expression in RAGE TG mice compared to controls when RAGE was overexpressed from E0–15.5 or E0–16.5. * *p* ≤ 0.05; blots were densitometrically normalized to β-actin and images are representative of three different immunoblotting experiments performed in quadruplicate.

FoxA2 is often a co-regulator with NKX2.1 in transcriptionally controlling target genes [17]. Because NKX2.1 was significantly decreased each day from E15.5 onward, we screened FoxA2 expression at the end of the pseudoglandular (E15.5) and start of the canalicular (E16.5) period of development (Figure 5). We observed significantly less FoxA2 expression at these important time points compared to non-transgenic controls (Figure 5).

### 3.4. Surfactant Protein C Was Down-Regulated in RAGE-Transgenic Mice

**Figure 6:**
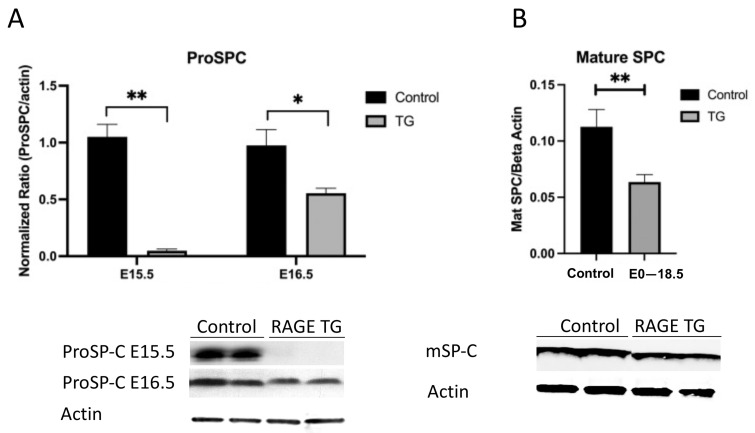
(**A**) Immunoblotting revealed a significant decrease in the expression of the SPC propeptide (Pro-SPC) in RAGE TG pup lungs treated with Dox from E0–E15.5 or E0–E16.5. * *p* ≤ 0.05 and ** *p* ≤ 0.01; blots were densitometrically normalized to β-actin and images are representative of three different immunoblotting experiments performed in quadruplicate. (**B**) Immunoblotting revealed significantly decreased elaboration of the mature SP-C protein when RAGE was induced from E0–E18.5 compared to controls. ** *p* ≤ 0.01; blots were densitometrically normalized to β-actin and images are representative of three different immunoblotting experiments performed in quadruplicate.

Because NKX2.1 and FoxA2 were both decreased in RAGE-transgenic mice, we hypothesized that their role in orchestrating alveolar cell differentiation was compromised. As such, we assessed the expression of the propeptide of surfactant protein C (ProSPC) synthesized by ATII cells, as well as the secreted mature peptide (Mature SPC). We discovered that ATII cells were likely impaired due to significantly less ProSPC in lysates during the period between the pseudoglandular and calicular periods (Figure 6A). Further, secreted mature SPC was markedly decreased in E18.5 TG lungs following RAGE over-expression (Figure 6B). Additional analyses of secreted mature SPB revealed no significant differences between double-transgenic and control pup lungs (not shown).

## 4. Discussion

This study reconfirmed significant pulmonary hypoplasia in mice when cells localized to the respiratory compartment over-express RAGE [4]. These and other related experimental pursuits reveal an interesting aspect of RAGE biology in that RAGE functions in diverse inflammatory settings [18,19,20], but may also provide a biological basis for lung embryogenesis as well. In the current undertaking, we observed a correlation between the over-expression of RAGE and the down-regulation of NKX2.1 and FoxA2. Diminished expression of NKX2.1, FoxA2, and a diversity of other key co-regulators leads to compromised differentiation of ATII cells that express surfactant proteins and other lung-specific products [21]. These and other molecular regulators normally contribute to a differentiation cascade that causes a cellular shift from the primitive pulmonary epithelium during early gestational periods toward an ATII phenotype essential for the postnatal lung. Such molecular orchestration during organogenesis causes ATII cells to proliferate, spatially organize, and express lung-specific genetic programs. As appropriate ATII quantities are reached, some differentiate under the control of NKX2.1 and FoxA2 into ATI cells that are absolutely critical during alveologenesis and the establishment of postnatal normal lung function [14,22]. In particular, NKX2.1 serves as a transcription factor critical in the formation of distal pulmonary structures and it specifically regulates surfactant protein genes that are important for the development of postnatal alveolar stability. Further, FoxA2 is a DNA-binding factor that critically influences endoderm formation via the activation of numerous target genes in concert with NKX2.1 [23]. Our discovery that pulmonary RAGE up-regulation causes diminished expression of these two critical transcription factors foreshadows the possibility that the expression of other genes under the control of NKX2.1 and FoxA2 involved in lung development may also be compromised [23,24].

An assumption that increased RAGE expression influences cell differentiation was reinforced by our observation that NKX2.1 and RAGE were colocalized in lung parenchyma and pulmonary expression of both NKX2.1 and FoxA2 were decreased in RAGE-transgenic mouse lungs. While not specifically tested in the current research, impaired cellular differentiation and lung patterning is not observed in RAGE knockout mice. For instance, alterations in lung morphology following branching morphogenesis and affected lung function in unstimulated RAGE knockout animals have not been observed. Differences in general lung architecture when comparing RAGE knockout and wild-type mice are imperceptible. These comparative observations suggest that redundant receptors may compensate for the absence of RAGE; however, increased availability of RAGE at critical periods of organogenesis appears to be sufficient for the permanent impairment of developmental milestones observed here. Subsequent queries into parallel RAGE pathways such as galectin-3 [25], Toll-like receptors 2 and 4 [26], or others may help to clarify such phenotypic considerations when RAGE is absent. A follow-up investigation is needed in order to assess the roles of these candidate parallel pathways during development.

An additional central finding of the current endeavor related to lung surfactant synthesis and secretion. Surfactants represent a complex collection of unique phospholipids and proteins. A vital function of surfactants is the reduction of surface tension observed at the pulmonary interface between air and liquid. Insufficient surfactant is a characteristic of premature infants that experience respiratory distress syndrome (RDS) [27]. While the objective of the current research was not to characterize RDS, our discovery that essential transcriptional regulators were decreased when RAGE was abundant reveals aberrant surfactant protein synthesis and secretion by ATII cells mediated at least in part by RAGE. Moreover, surfactant proteins are expressed in a cell-type-restricted manner, and the current research identified ATII cell compromise when RAGE is elevated (via pro-SPC assessment) as well as the secreted form of mature SPC. The promoter elements of surfactant proteins are activated following combinatorial interactions between multiple transcription factors, and previous research has identified NKX2.1 as a positive regulator for surfactant protein transcription [28].

In summary, elevated RAGE expression by alveolar epithelium results in developmental simplification potentially via NKX2.1 and FoxA2. While more research is required, differentially regulating RAGE expression may be a component of uncovering mechanistic programs that lead to insufficient lung tissue observed in cases of RDS or bronchopulmonary dysplasia (BPD). In fact, a limitation of this study is a lack of mechanistic detail as to how up-regulated RAGE expression causes decreased expression of NKX2.1 and FoxA2, two key transcriptional regulators. To what extent RAGE signaling intermediates crosstalk with signaling pathways that lead to NKX2.1/FoxA2 abundance would be a critical next step in elucidating such mechanisms. 

Clarification of the mechanisms that cause the phenotype observed in this model is needed. Further characterization may reveal that significant reduction of lung parenchyma is accompanied by fibrosis and a thickening of expanded alveoli observed in the BPD lung [29]. Further, RAGE expression is a known target of tobacco smoke exposure, and prenatal smoke exposure is also indicated in cases of lung simplification reminiscent of BPD [30]. Further evaluation of lung simplification following RAGE over-expression should therefore clarify imbalances between fibrosis and tissue damage in the context of impaired developmental events or tissue destruction. While the current research does not provide models for clinical diagnoses such as RDS or BPD, it provides an initial step in the identification of molecular targets that potentially contribute to such outcomes. 

## Data Availability

All data are presented within the article. Data and other materials are available from the corresponding author on reasonable request.
